# Extra-medullary recurrence of myeloid leukemia as myeloid sarcoma after allogeneic stem cell transplantation: impact of conditioning intensity

**DOI:** 10.1038/s41409-020-0984-4

**Published:** 2020-06-30

**Authors:** Jochen J. Frietsch, Friederike Hunstig, Christoph Wittke, Christian Junghanss, Tobias Franiel, Sebastian Scholl, Andreas Hochhaus, Inken Hilgendorf

**Affiliations:** 1 Klinik für Innere Medizin II, Hämatologie und Internistische Onkologie, Universitätsklinikum Jena, Jena, Germany; 2 I. Medizinische Klinik und Poliklinik, Universitätsklinikum Hamburg-Eppendorf, Hamburg, Germany; 3 Medizinische Klinik III, Klinik für Hämatologie, Onkologie und Palliativmedizin, Universitätsmedizin Rostock, Rostock, Germany; 4 Institut für Diagnostische und Interventionelle Radiologie, Universitätsklinikum Jena, Jena, Germany

**Keywords:** Haematological diseases, Medical research

## Abstract

Myeloid sarcoma (MS) as a solid extra-medullary (EM) manifestation of acute myeloid leukemia (AML), myeloproliferative or myelodysplastic syndromes is a rare presentation of relapse after allogeneic hematopoietic stem cell transplantation (HSCT). The databases of the Departments of Hematology and Oncology of the University Hospitals of Jena and Rostock were screened for patients aged 18 years or older for onset of MS after HSCT for myeloid malignancies between 2002 and 2019. Nineteen patients with MS were identified, the majority of whom had received reduced-intensity conditioning (RIC). The median onset of MS was 425 days after HSCT and the median overall survival since MS was 234 days. Although MS is associated with a poor prognosis, three patients survived more than two years and one more than 11 years after MS onset. These results indicate that RIC protocols may be associated with a higher risk of EM relapse. Since EM relapse occurred in the presence of Graft-versus-host-disease, these observations also demonstrate the limitations of graft-versus-tumor effects after HSCT. In conclusion, occurrence of MS after HSCT is associated with a poor prognosis, as multimodal curative concepts including intensive chemotherapy and another HSCT are often not viable.

## Introduction

Myeloid sarcoma (MS), also known as granulocytic sarcoma or chloroma, is defined as an extra-medullary (EM) and infiltrating tumor mass formation of blasts of one or more myeloid linages and maturating cells. It has been described as developing in isolation and de novo, as preceding systemic disease, or as a concomitant manifestation of acute myeloid leukemia (AML), myeloproliferative neoplasms (MPN) including blast phase chronic myeloid leukemia (CML), or myelodysplastic syndromes (MDS) [1–3]. It may also manifest as relapse, especially in recipients of allogeneic hematopoietic stem cell transplantation (HSCT) [2, 4–8]. MS can occur at any site in the body, e.g. in the central nervous system (CNS), skin, soft tissue, bones and testis with a slight preference for male sex [2, 6–9].

As diagnosis is challenging and requires a high level of suspicion, diagnostic workup should include tissue biopsy with immunohistochemistry, immunophenotyping, and genetic and molecular analysis. In addition, for the right selection of therapeutic modalities, bone marrow biopsy should be performed to exclude medullary involvement [10, 11]. Moreover, additionally performed Positron Emission Tomography and Computer Tomography allows for the evaluation of treatment response, reveals previously unnoticed manifestations, and increases diagnostic accuracy [12].

Although manifestation of MS is a rare event [13], 19 patients presenting with MS as a sign of relapse of primary disease after allogeneic HSCT were identified at the University Hospitals Jena and Rostock (Germany) between January 2002 and December 2019. A systematic review of the literature on EM relapse after HSCT and treatment available on PubMed is also provided.

## Methods

### Patient selection

The databases of the Departments of Hematology and Internal Oncology of the University Hospitals Jena and Rostock (Germany) were searched for patients with EM MS and carcinomatous meningitis after HSCT for AML, MPN, CML or MDS, and a retrospective review of individual medical records was performed. None of the patients analyzed had a previous history of MS bevor HSCT. Patients were included if they had histologically proven MS based on WHO criteria forming solid tumor masses [1, 3]. Patients with simple tissue infiltrations or effusions were excluded.

### Transplantation procedures

The majority of MS patients (16/19, 84.2%) received a reduced-intensity conditioning (RIC) based on treosulfan (11/19, 57.1%) or busulfan (4/19, 21.1%) in combination with fludarabine before undergoing allogenic HSCT [14–16]. The remaining MS patients received myeloablative conditioning (MAC; 2/19, 10.5%) with 12 Gy total body irradiation (TBI) in combination with cyclophosphamide [17], a non-myeloablative conditioning (NMAC; 1/19, 5.26%) with 2 Gy TBI and fludarabine [18], or a sequential regimen of clofarabine, cytarabine, busulfan and cyclophosphamide [19]. Graft-versus-host-disease (GvHD) prophylaxis consisted of cyclosporine A (CSA) in combination with short-term methotrexate (MTX) or mycophenolate mofetil (MMF), and anti-thymocyte globulin (ATG).

### Statistical analysis

Overall survival was calculated from the date of reinfusion of hematopoietic stem cells (OS^HSCT^) and from MS onset (OS^MS^) to date of death. MS free survival (MSFS) was defined as the time between HSCT and the date of histologically confirmed MS onset and only calculated for patients suffering from MS. In cases of repeated HSCTs, survival was calculated from the last HSCT. Differences between the Kaplan–Meier survival curves were evaluated by Log-rank test and those between frequencies of MS by the t-test with Welch’s correction. A *P* value of <0.05 was considered statistically significant. All analyses were conducted using GraphPad Prism 8.0.2 (GraphPad Inc.).

## Results

### Frequency and patient characteristics

Between January 1st, 2002 and December 31st, 2019, 307 patients with AML (289 patients with primary and 18 with secondary AML, sAML) underwent allogeneic HSCT at the University Hospital of Jena. The patients received MAC, NMAC, or RIC as follows: 103 (35.6%), 8 (2.8%) and 178 (61.6%) of patients with AML as well as 1 (5.6%), 0 (0%) and 17 (94.4%) of patients with sAML, respectively. MS after HSCT occurred in 16/307 (5.21%) of patients with AML. The total number of patients receiving HSCT was not assessed for the whole time period at the University Hospital of Rostock.

Altogether, 19 patients suffering from MS were identified. Patient characteristics are shown in Table 1. Median age of the 7 male and 12 female patients was 57 years (range: 28–65 years). At the time of HSCT, 14 patients were in first complete remission (CR), 4 in second CR, and 1 had histologically confirmed partial response. The majority of patients, 9/19 (47.4%), received HSCT from a matched-unrelated (MUD), 7/19 (36.8%) from a matched-related (MRD) and 3/19 (15.8%) from a mismatched unrelated (MMUD) donor.

**Table 1:** Patients’ characteristics.

No.	Age at disease onset [years]	Sex [m/f]	Diagnosis	Cyto-/ Molecular Genetics/ELN risk category [20]	Monoblastic morphology	Conditioning intensity [14–19]	Donor source	HCT-CI [50]	Severity of acute GvHD [22]	Severity of chronic GvHD [23]	Isolated extra-medullary MS	Chimerism at time of MS [%]	MSFS [days]	OS^MS^ [days]	Site of relapse [7, 33, 34]	Therapy for MS [antitumoral drugs, radiation, DLI]
1^a,b^	59	f	AML	Complex aberrant/adverse	No	RIC (bu, flu) [14]	MUD	2	I (skin II and liver I)	Mild	No (systemic relapse within 30 months)	100	1,021	4,052	Skin, breast [51], stomach, small intestine, right auricle, frontal sinus, intraspinally along spinal cord, carcinomatous meningitis	Intrathecal triple [32], LDAC [31]; radiation; DLI
2	55	f	AML	46,XX/FLT3-LM^high^, NPM1/intermediate	No	RIC (treo, flu) [15]	MMUD (C antigen)	5	III (intestinal III)	None	No (systemic relapse within 4 months)	79	134	234	Within brain mass [52], carcinomatous meningitis	Intrathecal triple [32], azacytidine [26]
3^b ^	57	f	AML	Complex aberrant/adverse	Yes	RIC (treo, flu) [15]	MRD	0	None	None	Yes	100	185	277	Submandibular gland, paravertebral soft tissue, vertebral body	LDAC [31], Mito-FLAG [24]; radiation
4	58	f	AML	46,XX/FLT3-LM^high^/adverse	Yes	RIC (treo, flu) [15]	MRD	4	None	Moderate	No (systemic relapse within 2 months)	Not analyzed	669	13	Muscle, pelvic bone, myocard	Quizartinib [28]
5^b ^	49	m	AML	Inversion 16, trisomy 22/intermediate	No	RIC (treo, flu) [15]	MRD	0	I (skin I)	Moderate	Yes	100	657	420	Pituitary gland [21, 53, 54], carcinomatous meningitis	Intrathecal triple [32]; radiation
6^a,c^	59	m	AML	Inversion 16, trisomy 22/KMT2A rearrangement/adverse	Yes	NMAC (flu, 2 Gy TBI) [18]	MUD	0	II (skin III)	None	Yes	100	236	178	Within spinal cord tissue, carcinomatous meningitis	Intrathecal triple [32], IDAC [25]
7	54	m	AML	del 5q, trisomy 13/FLT3-LM^high^/adverse	No	RIC (treo, flu, cytarabine) [16]	MUD	0	None	Moderate	No (systemic relapse within 12 months)	88	442	990	Within brain mass [52], carcinomatous meningitis	Intrathecal triple [32], Mito-FLAG [24], LDAC [31], mitoxantrone [29], azacytidine [26]; sorafenib [27]; radiation; DLI
8	42	f	sAML	del 5q/adverse	No	RIC (bu, flu) [14]	MRD	1	III (intestinal III)	None	Yes	100	539	141	Epidural along spinal cord, pleura, skin	Radiation
9	61	m	sAML	del 20, trisomy 21/intermediate	No	RIC (clofarabine, cytarabine, bu, cy) [19]	MUD	1	II (skin III)	None	No (systemic relapse within 45 months)	100	855	858	Skin, lung	LDAC [31], mitoxantrone [29], azacytidine [26]; radiation
10	58	f	sAML	del 5q/adverse	No	RIC (treo, flu) [15]	MUD	3	None	None	Yes	100	425	83	Lung, breast [51], skin	LDAC [31], azacytidine [26]
11^b^	29	m	sAML	Trisomy 8/intermediate	No	RIC (bu, flu) [14]	MMUD (B and DR antigen)	0	IV (skin IV and liver II)	Severe	Yes	100	1,276	286	Thoracic wall, orbital cavity	LDAC [31], radiation; sorafenib [27]; radiation
12	59	f	sAML	t(8;16)(p11;q13)/intermediate	Yes	RIC (bu, flu) [14]	MUD	3	None	Mild	No (systemic relapse within 5 months)	96	136	154	Skin	LDAC [31], etoposide [29]; radiation
13^d^	36	f	AML	t(8;21)(q22;q22), trisomy 8/RUNX1-RUNX1T1; FLT3-LM^low^/intermediate	Yes	RIC (treo, flu) [15]	MUD	3	None	None	Yes	100	202	21	Subdural and epidural at the posterior cranial fossa, carcinomatous meningitis	Intrathecal triple [32]
14	28	m	AML	t(10;11)(p11;q11)/KMT2A-MLLT10/adverse	No	MAC (cy, 12 Gy TBI) [17]	MRD	0	None	None	No (systemic relapse within 11 months)	31	346	295	Within spinal cord tissue, inner ear, facial nerve canal [55, 56], carcinomatous meningitis	Intrathecal triple [32], IDAC [25]
15	44	f	sAML	45,XX, del7 /adverse	No	MAC (cy, 12 Gy TBI) [17]	MRD	3	III (skin)	None	No (systemic relapse within 6 months)	6	281	18	carcinomatous meningitis	Intrathecal triple [32], IDAC [25], ETI [30]; DLI
16	63	f	AML	46,XX/FLT3-LM^high^/adverse	No	RIC (treo, flu) [15]	MMUD (C antigen)	2	None	Moderate	No (systemic relapse within 16 months)	Not assessed	489	36	Parietal and occipital meninges, carcinomatous meningitis	Intrathecal triple [32], IDAC [25]; decitabine [26]
17^a,e^	65	f	sAML	Complex aberrant/adverse	Yes	RIC (treo, flu) [15]	MUD	1	None	None	Yes	100	80	42	Skin, within brain [52], bone, muscle	Mitoxantrone [29]; radiation
18^a,c,e^	44	f	sAML	Not assessed	Yes	RIC (treo, flu) [15]	MRD	Not assessed	I (skin II)	Severe	Yes	100	405	318	Muscle, vertebral body	LDAC [31], decitabine [26]; radiation
19^e^	61	m	sAML	46,XY,+1,der (1;7)(q10;p10)/CEBPA, JAK2, RUNX1/adverse	No	RIC (treo, flu) [15]	MUD	2	None	None	No (systemic relapse within 12 months)	75	Alive	Alive	Vertebral body	Mito-FLAG [24]

*AML* acute myeloid leukemia, *bu* busulfan, *CR* complete remission, *cy* cyclophosphamide, *DLI* donor lymphocyte infusion, *FLT3-LM*^*high*^ allelic ratio ≥ 0.5, *FLT3-LM*^*low*^ allelic ratio < 0.5, *GvHD* Graft-versus-host-disease, *HSCT* hematopoietic stem cell transplantation, *IDAC* intermediate-dose cytarabine, *Intrathecal triple* methotrexate, cytarabine, dexamethasone, *LDAC* low-dose cytarabine subcutaneous, *MAC* myeloablative conditioning, *MMUD* mismatched unrelated donor, *MRD* matched-related donor, *MS* myeloid sarcoma, *MSFS* MS free survival, *MUD* matched-unrelated donor, *NMAC* non-MAC, *OS*^*MS*^ overall survival since MS onset, *PR* partial response, *RIC* reduced-intensity conditioning, *TBI* total body irradiation, *treo* treosulfan, *sAML* secondary AML.

^a^Transplanted in second CR.

^b^See Supplementary Materials; for patient #1, #3, #5 and #11 see Supplementary Figs. S1, S2, S3 and S4, respectively.

^c^Second HSCT.

^d^Transplanted in PR.

^e^Transplanted at the University Hospital of Rostock.

Furthermore, 2/37 (5.41%) patients with CML and MS after HSCT were identified, but excluded from analysis.

### Cytogenetics

Cytogenetic analysis of bone marrow was available for 18 patients and is given in Table 1. FLT3-LM mutation was confirmed in 4 patients at a high (≥0.5) and in 1 patient with a low allelic ratio (<0.5). AML patients were classified with intermediate (6/19, 31.6%) or adverse risk (12/19, 63.2%) according to ELN guidelines [20] (see Table 1). No patient was classified with favorable risk, and one patient was not classifiable, as cytogenetic and molecular genetics were not assessable.

### Manifestation of myeloid sarcoma

The median onset of MS was 425 days after HSCT (MSFS; range: 80–1276 days). 9/19 (47.4%) relapsed in EM sites only, and 5/19 (26.3%) relapsed in both EM sites and the bone marrow at the time of MS onset. Three patients (26.3%) with EM relapse progressed to bone marrow involvement within a median time of 5 months (range: 2–24 months) after MS onset. Two patients sustained EM after medullary relapse (6 and 18 months), one of whom had initially achieved CR after medullary relapse using FLT3-inhibition.

The regions and organs affected are summarized in Table 1 and vary from very typical tissues such as skin, bone or lymph node to extremely rare presentations of MS affecting the pituitary gland [21], breast tissue, eye socket, thoracic wall, atrium, intestine, paranasal sinuses, inner ear, and facial nerve canal. Interestingly, 13/19 (68.4%) patients developed MS despite a history of GvHD [5 patients with acute, 4 with chronic, and 4 with acute GvHD (aGVHD) becoming chronic/overlap]. Acute and chronic GvHD (cGvHD) was classified according to the criteria of Glucksberg et al. [22] and Filipovich et al. [23], respectively.

### Treatment

MS was treated based on the pattern of leukemic involvement. In case of EM relapse, local applicable anti-tumor treatment methods were used. Local radiotherapy was applied in 11/19 (57.9%) patients as either the sole treatment modality or in combination. Based on the slow disease kinetics and a drop of chimerism, 3/19 patients (15.8%) received infusion of donor lymphocytes. Three patients were treated with re-induction therapy with Mito-Flag [24] and four patients with intermediate-dose cytarabine [25]. Six patients received epigenetic therapy (6/19, 31.6%) [26]. Two patients with FLT3-LM-positive AML were treated with sorafenib [27] and one with quizartinib [28]. Etoposide and a combination of etoposide, 6-thioguanine, and idarubicine were applied in one case each [29, 30]. The majority of patients (14/19, 73.7%) received additionally, intermittently or sequentially low-dose cytarabine or mitoxantrone [29, 31]. Nearly half of patients (9/19, 47.4%) suffering from concomitant carcinomatous meningitis were treated with intrathecal triple therapy [32]. Surgical tumor resection was not performed in any case.

### Outcome and survival

MS occurred within a median of 425 days after HSCT. 18/19 patients died: 13/19 (68.4%) patients from progression of MS or the underlying systemic disease and 5/19 (26.3%) patients from severe infections.

**Fig. 1:**
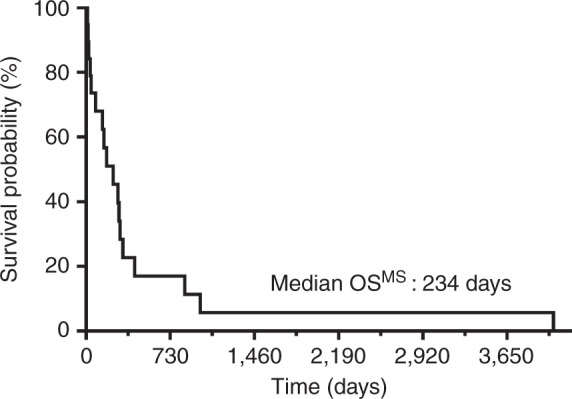
Kaplan-Meier survival curve showing overall survival from MS onset (OS^MS^) of all 19 patients. Median OS^MS^ was 234 days.

Median OS^HSCT^ was 641 days and OS^MS^ 234 days (Fig. 1). OS^MS^ did not differ significantly in patients suffering from sAML compared to primary AML (not shown). Therefore, the entities AML and sAML were combined to compare the frequency of MS development when treated with MAC and lower intensity conditioning (LIC; RIC and NMAC), revealing significantly more MS in the LIC-treated AML and sAML cohort (*P* = 0.024, unpaired t-test with Welch’s correction, not shown).

Survival probabilities were significantly better in GvHD patients (MSFS *P* = 0.048, OS^HSCT^ *P* = 0.032, not shown; Log-rank). Additionally, patients with non-monoblastic differentiation had a significantly longer survival (MSFS *P* = 0.022, OS^HSCT^ *P* = 0.021, OS^MS^ *P* = 0.071, not shown; Log-rank). Other variables being analyzed, such as sex, risk stratification according to ELN-criteria [20], isolated EM versus systemic relapse and MS with regard to affection of CNS did not affect OS or MSFS. Notably, affection of CNS did not divide patients in to distinct groups.

## Discussion and conclusion

Isolated EM relapse after HSCT is a rare event, with incidence rates between 0.65–30% [13, 33–36] and a median MSFS of 210 days [6, 34]. Three-fourths of EM relapses happened within the first 2 years after HSCT [7]. In the largest series of isolated MS after HSCT, OS at 5 years was 48% and disease-free survival 36% [37]. Although the incidence rate of MS of 16/307 (5.21%) within 18 years after HSCT and median OS^MS^ with 234 days is in line with previous reports, a median OS^HSCT^ of 641 days is not that adverse [36, 38–41]. With the exception of three patients, the reported results confirm the finding that only patients relapsing within 1 year after HSCT suffered from concomitant EM and systemic relapse [13, 42]. However, as 8–20% and even up to 95% of patients progress from isolated EM to systemic relapse after HSCT, the rate at our clinic was low [33, 35]. Nonetheless, relapse is a major cause of mortality [43, 44].

The engrafting immune system after HSCT is held to be responsible for inducing remission and long-term survival by eliminating the malignant cells. Alloreactive T cells are thought to be responsible not only for the graft-versus-leukemia (GvL) effect but also for mediating GvHD [43, 44].

Previous cGvHD was associated with better OS [10] and aGvHD with better relapse-free survival [13]. Thus, OS^HSCT^ and MSFS are higher in GvHD patients, compared to those without GvHD at all. However, in contrast to a previous study [42], the association between cGvHD before relapse and improved OS after relapse could not be confirmed. Since nearly half of the patients (9/19; 47.4%) with EM relapse did not develop systemic relapse, the relevance of GvL must be stressed.

Beyond GvHD, further factors have been identified as being associated with EM relapse after HSCT, including donor lymphocyte infusion, younger age, EM manifestations before HSCT, advanced disease at HSCT, unfavorable cytogenetics, and M4/M5 subtypes according to FAB classification [13, 35]. No association between genetic risk stratification according to ELN-criteria [20] and MSFS and OS could be confirmed. Nevertheless, survival was significantly better for patients with non-monoblastic differentiation. In addition, the intensity of conditioning seems to play a role in the onset of EM relapse after HSCT. An association between EM relapse and busulfan-based regimens was observed [7, 45]. Taking into account that only two patients developed MS after MAC in contrast to all other patients after LIC (*P* = 0.024, unpaired t-test, with Welch’s correction, not shown), suggests that LIC may increase the risk for EM relapse.

Notably, except for one patient (1/9) whose clinical condition deteriorated quickly before diagnostic imaging, we were able to prove that carcinomatous meningitis was concomitant to solid involvement of the CNS in every single case. Therefore extensive and enlarged diagnostic imaging of the whole CNS is recommended as part of the diagnostic workup in order to reveal solid MS manifestations.

Although the armamentarium for the treatment of isolated MS has been widened since the introduction of targeted therapies, one should keep in mind that MS represents part of a systemic disease and is believed to progress rapidly to systemic relapse without therapy [9, 11]. Therefore, systemic chemotherapy may be an appropriate therapeutic approach [34, 46]. Nevertheless, the optimal therapy post-HSCT is a matter of debate. The choice of therapy depends on the time from transplant to MS onset, the patients’ general health condition, the chimerism, and the presence of GvHD [10]. As a less effective GvL-effect is considered to be causative for the formation of MS, donor lymphocyte infusion and tapering of immunosuppression are recommended [6–8, 10]. Hypomethylating agents might enhance GvL by increasing HLA and tumor-associated antigen expression [13, 35, 47]. Another therapeutic option is the application of gemtuzumab ozogamizin [13, 35]. Whenever suitable, use of targeted therapies, i.e. tyrosine kinase inhibitors for FLT3-ITD, is recommended [11, 47]. In addition, inhibition of CTL-4 may prevent immune escape and can result in complete response of MS [48]. In case of rapid tumor growth, surgery or palliative radiotherapy can be considered [9, 11]. A combinational approach seems to be advantageous [6, 7, 9].

Although, HSCT is a feasible option for the treatment of isolated or leukemic MS, the impact of a secondary allogeneic HSCT, remains unclear [10, 35, 37, 46, 49].

In this reported cohort, the majority of patients received local or subcutaneous applicable anti-tumor therapy, e.g. epigenetic drugs, to facilitate outpatient treatment in a predominantly palliative situation. Patient #1 underscores particularly well the importance of a wise choice of the therapeutic strategy that was most likely the cause of this patient’s over 11-year-suvival with MS (see Table 1, Supplementary Materials and Supplementary Fig. S1; additional information concerning patient #3, #5 and #11 is also provided within the Supplementary Materials and Supplementary Figs. S2–S4). Although this is an individual case, it emphasizes that despite the very poor prognosis recurrent isolated MS can result in long-term survival [33]. Au and colleagues even reported prolonged survival in up to a third of all patients [40].

The limitations of the study are the retrospective design and the small number of patients. However, it demonstrates that (a) the intensity of conditioning may have an impact on the onset of MS and that (b) a choosing treatment wisely can lead to a prolonged OS in an utmost dismal situation. Future studies are necessary to further elucidate the pathophysiological interconnections, discover novel therapeutic agents and finally improve the outcome of HSCT recipients with MS.

## Supplementary information

Supplemental Material

## Data Availability

The authors confirm that the data supporting the findings of this study are available within the article.
