# Supplementary material for: Extra-medullary recurrence of myeloid leukemia as myeloid sarcoma after allogeneic stem cell transplantation: impact of conditioning intensity
Source: Bone Marrow Transplant. 2020 Jun 30;56(1):101–9. doi: 10.1038/s41409-020-0984-4 (PMC7796857; doi:10.1038/s41409-020-0984-4)
Supplement: Supplementary file 1 — Supplemental Material [file 41409_2020_984_MOESM1_ESM.pdf]

Supplemental Material

Extra-medullary recurrence of myeloid leukemia as myeloid sarcoma after allogeneic stem cell transplantation: impact of conditioning intensity

Jochen J Frietsch, Friederike Hunstig, Christoph Wittke, Christian Junghanss, Tobias Franiel, Sebastian Scholl, Andreas Hochhaus, Inken Hilgendorf

Supplemental Case presentations

Supplemental Case #1 (patient #1)

A 59 year-old female was diagnosed with acute myeloid leukemia (AML), M2 according to FAB classification (FAB M2), in 2001. Cytogenetic workup revealed an additional chromosome 11, 47,XX. She received induction and consolidation I chemotherapy according to the protocol of the German AML Intergroup [25]. For consolidation II, idarubicin was replaced by mitoxantrone. She achieved complete remission (CR) but suffered from early relapse four months later. Salvage chemotherapy with cytarabine and cyclophosphamide was administered.

After reduced intensity conditioning (RIC), she received allogeneic hematopoietic stem cell transplantation (HSCT) from a male matched unrelated donor (MUD) in May 2002. While tapering immunosuppression, she developed mild chronic Graft-versus-host-disease (cGvHD). Immunosuppression was stopped 1,021 days after HSCT due to histologically proven manifestation of a myeloid sarcoma (MS) of the scalp with a diameter of 20 mm. She had a complete donor chimerism. MS was treated with fractionated local irradiation therapy (36 Gy). In August 2005 MS of the scalp re-occurred with histologically proven involvement of cervical lymph nodes.

Bone marrow biopsy with mixed chimerism of 61% led to administration of five cycles of subcutaneous cytarabine and 6-tioguanine followed by two infusions of donor lymphocytes (DLI) resulting in CR and enduring 100% donor chimerism. A quarter of a year later cGvHD worsened again, and immunosuppression was escalated gradually by prednisolone, mycophenolate mofetil (MMF), everolimus, PUVA therapy and extracorporeal photophoresis (ECP). More than 3.5 years after the last DLI, mammography revealed a 32 x 21 x 20 mm nodule in the right breast (Supplemental Figure S1A). Subsequently, segmental resection was performed. Pathohistological workup confirmed another manifestation of MS that was treated with fractionated local irradiation (26 Gy). Another year later a 40 x 35 mm solid MS of the stomach was histologically confirmed (Supplemental Figure S1B + C). After fractionated local irradiation, routine mammography yielded MS in the left breast which was irradiated after histological confirmation. Four months later she suffered from relapse of MS in the left breast which was irradiated once more. In May 2012, she underwent segmental resection of the jejunum because of intestinal obstruction caused by MS. Due to MS and concomitant cGvHD, ECP finally enabled complete tapering of immunosuppressive therapy. Although there was complete donor chimerism, she received three courses of subcutaneous cytarabine [31]. Arrhythmia, probably caused by a solid swelling of the right auricle, led to pacemaker implantation one year later. However, she did not permit histological examination. In addition, a CT-scan revealed a solid tumour in the maxillary sinus (Supplemental Figure S1D), which turned out to be another MS manifestation requiring irradiation. Four courses of subcutaneous cytarabine [31] were administered upon progression with newly occurring tumour manifestations on the distal fibula, increasing size of the heart-MS with concomitant pleural effusions since March 2015. A quarter of a year later, she complained of unsteady gait,

urinary incontinence, constipation and numbness. She received intrathecal triple therapy [32] due to carcinomatous meningitis with intradural manifestations at cervical vertebral body (VB) 7, thoracic VB 1 and lumbar VB 4 and 5, and along the spinal cord. Finally, she received irradiation of the spinal MS and died 4,052 days after the first manifestation of MS.

Supplemental Case #2 (patient #3)

This 57-year-old female was diagnosed with AML (FAB M4). Cytogenetics revealed a complex karyotype. After induction and consolidation chemotherapy with Ida-IDAC [25], allogeneic HSCT was performed after RIC [15]. Six months later, she was in CR of AML with complete donor chimerism but complained of a 7 x 2 mm painful swelling of the submandibular gland (Supplemental Figure S2A). Histology revealed a manifestation of MS. Immunosuppression was stopped and radiotherapy initiated. However, she developed liver GvHD and received immunosuppression with tacrolimus. Within six months after manifestation of the MS, she presented with pains of the lumbar spine radiating to the right leg. Magnetic resonance imaging (MRI) revealed multifocal tumour manifestations of spine with extra-osseous involvements (Supplemental Figure S2B). A further manifestation of MS was confirmed by histology. She received cytoreductive therapy with intravenous cytarabine (100 mg/m^2 days 1-3) and continued with fludarabine (15 mg/m^2, BID, on days 1-5), cytarabine (1,000 mg/m^2, BID, on days 1-5) and mitoxantrone (7 mg/m^2, on days 1, 3 and 5) [24]. 277 days after diagnosis of MS she died from candidemia with multi-organ failure.

Supplemental Case #3 (patient #5)

This 49-year-old male, diagnosed with AML, M2 according to FAB classification, with trisomy 22 and inversion 16 received induction and consolidation chemotherapy with Ida-IDAC and two further courses of consolidation [25]. 657 days after HSCT from a MRD after RIC [15], he had complete donor chimerism and no signs of cytological relapse of AML. However, he complained of headache, vertigo, attacks of sweating, visus decrease, polydipsia and progressive erectile dysfunction. MRI revealed an enlargement of the pituitary gland (Supplemental Figure S3, see also Fleischmann et al. [21]). Histological workup of stereotaxic biopsy confirmed manifestation of MS and radiation of the pituitary was started. He received 22 cycles of intrathecal triple therapy [32] due to carcinomatous meningitis and died 420 days after diagnosis of MS from severe pulmonary embolism.

Supplemental Case #4 (patient #11)

This 29-year-old male was initially diagnosed with myelodysplastic syndrome (MDS) with excess of blasts 2 with transition to secondary AML (FAB M4). Due to insufficient blast clearance following Ida-IDAC induction [25], salvage therapy with cytarabine and cyclophosphamide was administered. He received HSCT from a mismatched unrelated donor (MMUD) after conditioning with busulfan and fludarabine [14] in 1st CR of sAML. While tapering immunosuppressive therapy, the patient developed grade II cutaneous and grade I hepatic acute GvHD, so immunosuppression had to be re-intensified. Although GvHD meanwhile deteriorated to grade IV, chimerism continued dropping to 31%. Finally, continuous administration of intravenous cytarabine for 3 days resulted in complete donor chimerism. Despite chronic GvHD, a CT-supported biopsy confirmed MS of the thoracic wall 1,276 days after HSCT in Janu-

ary 2010 (Supplemental Figure S4A). In spite of radiotherapy, MS progressed and new manifestations occurred in the right eye socket (Supplemental Figure S4B) and the apex of the lung, which were irradiated. Although MS responded to cytarabine (20 mg, BID, on days 1-10, 5 cycles) [31] in combination with 200 mg sorafenib twice daily [27], the patient died 286 days after manifestation of MS.

Supplemental Figure Lengends

Supplemental Figure S1: Illustration of patient #1 sites of affection

Horizontal T2-weighted magnetic resonance imaging of the breast (A) showing a suspicious tumor with a dimension of 32 x 21 x 20 mm; horizontal computed tomography of the upper abdomen (B) and the paranasal sinus (D), revealing a 13 mmm swelling of the gastric wall and complete obstruction of the maxillary sinus; gastroscopy (C) revealed a 50 mm tumor at the greater curvature of the stomach.

Supplemental Figure S2: MRI of the soft tissue of the neck and the spine of patient #3

Coronary T2-weighted magnetic resonance imaging of the soft tissues of the neck (A), demonstrating a swelling and a fluid border enclosing the submandibular gland, sagittal T1-weighted magnetic resonance imaging of the spine (B), demonstrating several extra-osseous tumors.

Supplemental Figure S3: Imaging of patient #5 pituitary

Sagittal brain magnetic resonance imaging (A), T1-weighted image showing enlargement and contrast agent uptake of the pituitary.

Supplemental Figure S4: Illustration of patient #11 pattern of involvement

Horizontal (A) computed tomography of the chest, yielding intrapulmonary, pleural and retroperitoneal manifestations of MS, T1-weighted magnetic resonance imaging of the head, revealing a 27 x 22 x 16 mm tumor of the orbit in contact with the lacrimal gland (B).

Supplemental Figures

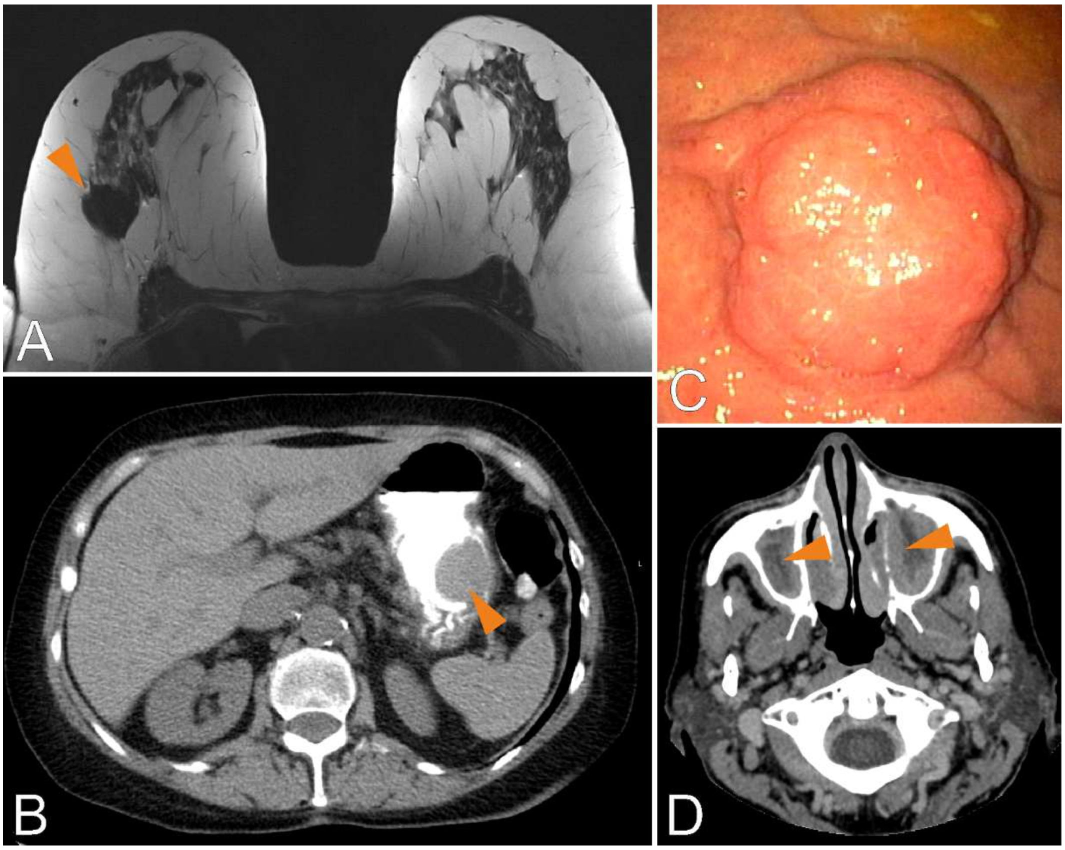

Supplemental Figure S1

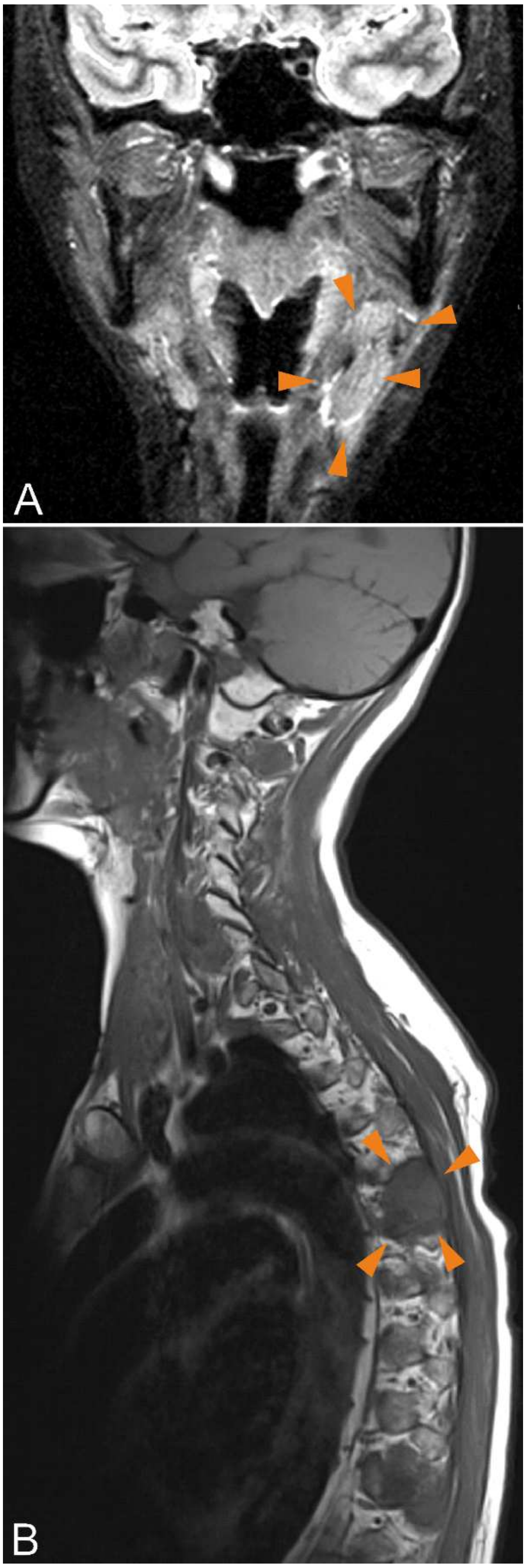

Supplemental Figure S2

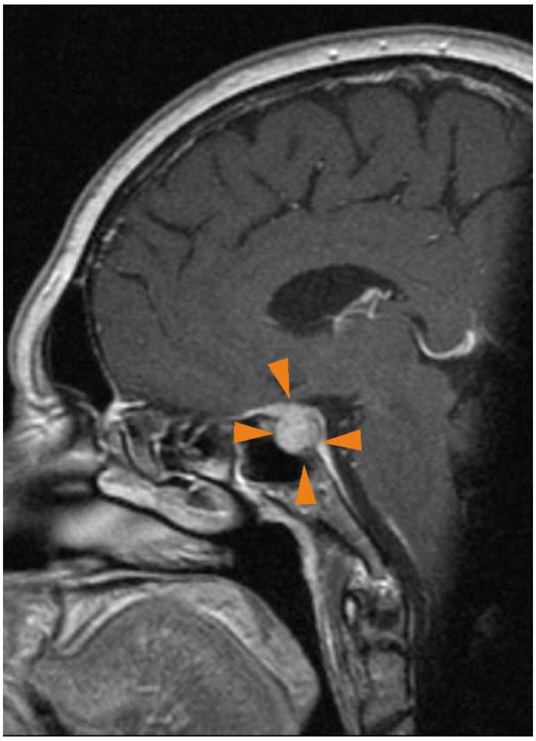

Supplemental Figure S3

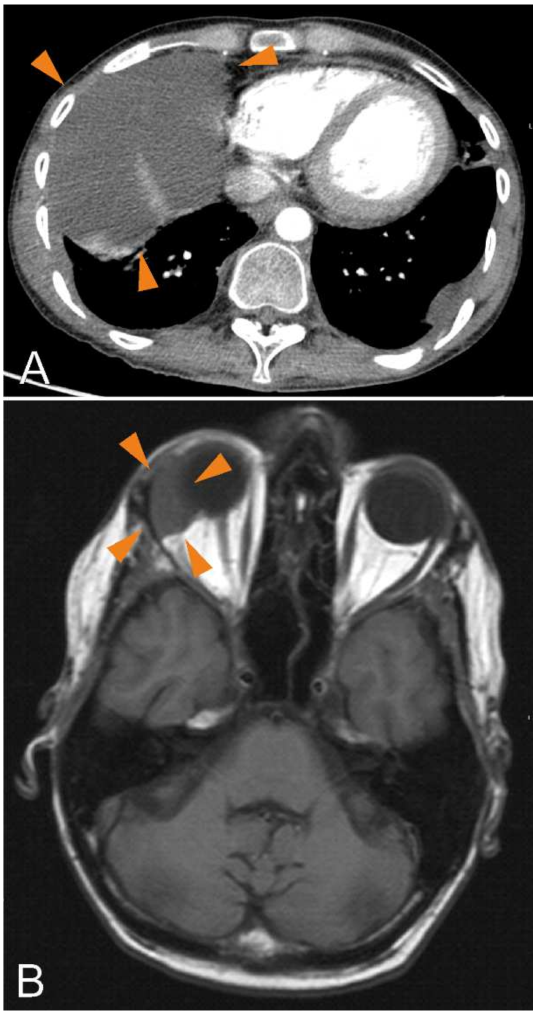

Supplemental Figure S4
